# Supplementary material for: COZOID: contact zone identifier for visual analysis of protein-protein interactions
Source: BMC Bioinformatics. 2018 Apr 6;19:125. doi: 10.1186/s12859-018-2113-6 (PMC5889581; doi:10.1186/s12859-018-2113-6)
Supplement: Supplementary file 2 — Software build. Executable binary file of the software tool. (ZIP 118,784 kb) [file 12859_2018_2113_MOESM2_ESM.zip › cozoid/java/docs/beansbinding-1.2.1-doc.zip!/org/jdesktop/el/package-summary.html]

org.jdesktop.el (Beans Binding)


---


|  |  |  |
| --- | --- | --- |
| **Overview** \| **Package** \| Class \| **Use** \| **Tree** \| **Deprecated** \| **Index** \| **Help** | |  |
| **PREV PACKAGE**   **NEXT PACKAGE** | **FRAMES**    **NO FRAMES**     **All Classes** |  |


---

## Package org.jdesktop.el

Customized version of EL for Beans Binding; not for general use.

**See:**
  
          **Description**

| **Interface Summary** | |
| --- | --- |
| **ELContextListener** | The listener interface for receiving notification when an `ELContext` is created. |

| **Class Summary** | |
| --- | --- |
| **ArrayELResolver** | Defines property resolution behavior on arrays. |
| **BeanELResolver** | Defines property resolution behavior on objects using the JavaBeans component architecture. |
| **BeanELResolver.BeanProperties** |  |
| **BeanELResolver.BeanProperty** |  |
| **CompositeELResolver** | Maintains an ordered composite list of child `ELResolver`s. |
| **ELContext** | Context information for expression evaluation. |
| **ELContextEvent** | An event which indicates that an `ELContext` has been created. |
| **ELResolver** | Enables customization of variable and property resolution behavior for EL expression evaluation. |
| **Expression** | Base class for the expression subclasses `ValueExpression` and `MethodExpression`, implementing characterstics common to both. |
| **Expression.ResolvedProperty** |  |
| **Expression.Result** |  |
| **ExpressionFactory** | Parses a `String` into a `ValueExpression` or `MethodExpression` instance for later evaluation. |
| **FunctionMapper** | The interface to a map between EL function names and methods. |
| **ListELResolver** | Defines property resolution behavior on instances of `List`. |
| **MapELResolver** | Defines property resolution behavior on instances of `Map`. |
| **MethodExpression** | An `Expression` that refers to a method on an object. |
| **MethodInfo** | Holds information about a method that a `MethodExpression` evaluated to. |
| **ResourceBundleELResolver** | Defines property resolution behavior on instances of `ResourceBundle`. |
| **ValueExpression** | An `Expression` that can get or set a value. |
| **VariableMapper** | The interface to a map between EL variables and the EL expressions they are associated with. |

| **Enum Summary** | |
| --- | --- |
| **Expression.Result.Type** |  |

| **Exception Summary** | |
| --- | --- |
| **ELException** | Represents any of the exception conditions that can arise during expression evaluation. |
| **MethodNotFoundException** | Thrown when a method could not be found while evaluating a `MethodExpression`. |
| **PropertyNotFoundException** | Thrown when a property could not be found while evaluating a `ValueExpression` or `MethodExpression`. |
| **PropertyNotWritableException** | Thrown when a property could not be written to while setting the value on a `ValueExpression`. |

## Package org.jdesktop.el Description

Customized version of EL for Beans Binding; not for general use.
It is expected that we'll sync up with an official version of EL
in the future.

Provides the API for the **Unified Expression Language** shared by
the JSP 2.1 and JSF 1.2 technologies.

The Expression Language (EL) is a simple language designed to satisfy
the specific needs of web application developers. It is currently defined
in its own specification document within the JavaServer Pages (tm) (JSP)
2.1 specification, but does not have any dependencies on any portion
of the JSP 2.1 specification. It is intended for general use outside of
the JSP and JSF specifications as well.

This package contains the classes and interfaces that describe
and define the programmatic access to the Expression Language engine. The API
is logically partitioned as follows:

- EL Context
- Expression Objects
- Creation of Expressions
- Resolution of Model Objects and their Properties
- EL Functions
- EL Variables

### EL Context

An important goal of the EL is to ensure it can be used in
a variety of environments. It must therefore provide enough flexibility
to adapt to the specific requirements of the environment where it is
being used.

Class `javax.el.ELContext` is what links
the EL with the specific environment where it is being used.
It provides
the mechanism through which all relevant context for creating or
evaluating an expression is specified.

Creation of `ELContext` objects is controlled through
the underlying technology. For example, in JSP, the
`JspContext.getELContext()` factory method is used.

Some technologies provide the ability to add an `javax.el.ELContextListener`
so that applications and frameworks can ensure their own context objects
are attached to any newly created `ELContext`.

### Expression Objects

At the core of the Expression Language is the notion of an *expression*
that gets parsed according to the grammar defined by the Expression Language.

There are two types of expressions defined by the EL: *value expressions*
and *method expressions*. A `javax.el.ValueExpression` such as
`"${customer.name}"` can be used either
as an *rvalue* (return the value associated with property `name`
of the model object `customer`) or as an *lvalue*
(set the value of the property `name` of the model object
`customer`).

A `javax.el.MethodExpression` such as
`"${handler.process}"` makes it possible to invoke a method
(`process`) on a specific model object (`handler`).

All expression classes extend the base class `javax.el.Expression`, making them
serializable and forcing them to implement `equals()` and
`hashCode()`. Morevover, each method on these expression classes
that actually evaluates an expression receives a parameter
of class `javax.el.ELContext`,
which provides the context required to evaluate the expression.

### Creation of Expressions

An expression is created through the `javax.el.ExpressionFactory` class.
The factory provides two creation methods; one for each type of expression
supported by the EL.

To create an expression, one must provide an `javax.el.ELContext`,
a string representing
the expression, and the expected type (`ValueExpression`) or signature
(`MethodExpression`).
The `ELContext` provides the context necessary to parse an expression.
Specifically, if the expression uses an EL function
(for example `${fn:toUpperCase(customer.name)}`) or an
EL variable, then
`javax.el.FunctionMapper` and `javax.el.VariableMapper`
objects must be available within the `ELContext` so that EL functions and
EL variables are properly mapped.

### Resolution of Model Objects and their Properties

Through the `javax.el.ELResolver` base class, the EL
features a pluggable mechanism
to resolve model object references as well as properties of these objects.

The EL API provides implementations of `ELResolver` supporting
property resolution for common data types which include
arrays (`javax.el.ArrayELResolver`), JavaBeans (`javax.el.BeanELResolver`), `List`s (`javax.el.ListELResolver`),
`Map`s (`javax.el.MapELResolver`), and `ResourceBundle`s (`javax.el.ResourceBundleELResolver`).

Tools can easily obtain more information about resolvable model objects and their
resolvable properties by calling
method `getFeatureDescriptors` on the `ELResolver`. This method exposes objects
of type `java.beans.FeatureDescriptor`, providing all information of interest
on top-level model objects as well as their properties.

### EL Functions

If an EL expression uses a function
(for example `${fn:toUpperCase(customer.name)}`), then a
`javax.el.FunctionMapper`
object must also be specified within the `ELContext`.
The `FunctionMapper` is responsible to map
`${prefix:name()}` style functions to
static methods that can execute the specified functions.

### EL Variables

Just like `javax.el.FunctionMapper` provides
a flexible mechanism to add functions to the EL, `javax.el.VariableMapper`
provides a flexible mechanism to support the notion of
**EL variables**.

An EL variable does not directly refer to a model object that can then
be resolved by an `ELResolver`. Instead, it refers to an EL
expression. The evaluation of that EL expression gives the EL variable
its value.

For example, in the following code snippet
> `<h:inputText value="#{handler.customer.name}"/>`

`handler` refers to a model object that can be resolved by an EL Resolver.

However, in this other example:
> ```
> <c:forEach var="item" items="#{model.list}">
>    <h:inputText value="#{item.name}"/>
> </c:forEach>
> ```

`item` is an EL variable because it does not refer directly to a model
object. Instead, it refers to another EL expression, namely a
specific item in the collection referred to by the EL expression
`#{model.list}.`

Assuming that there are three elements in `${model.list}`, this means
that for
each invocation of `<h:inputText>`, the following information
about `item` must be preserved in the `javax.el.VariableMapper`:
> first invocation: `item` maps to first element in `${model.list}`  
> second invocation: `item` maps to second element in `${model.list}`  
> third invocation: `item` maps to third element in `${model.list}`

`VariableMapper` provides the mechanisms required to allow the mapping
of an EL variable to the EL expression from which it gets its value.

---


|  |  |  |
| --- | --- | --- |
| **Overview** \| **Package** \| Class \| **Use** \| **Tree** \| **Deprecated** \| **Index** \| **Help** | |  |
| **PREV PACKAGE**   **NEXT PACKAGE** | **FRAMES**    **NO FRAMES**     **All Classes** |  |


---
